# Supplementary material for: Identifying effective interventions to promote consumption of protein-rich foods from lower ecological footprint sources: A systematic literature review
Source: PLOS Glob Public Health. 2022 Mar 16;2(3):e0000209. doi: 10.1371/journal.pgph.0000209 (PMC10021177; doi:10.1371/journal.pgph.0000209)
Supplement: Attachment — Submitted filename: Response to Reviewers.docx [file pgph.0000209.s002.docx]

| Editor and Reviewers’ comments | Authors’ response | Location of corrections |
| --- | --- | --- |
| Editor | | |
| 1. Please amend your detailed Financial Disclosure statement. This is published with the article, therefore should be completed in full sentences and contain the exact wording you wish to be published.  i) Please include all sources of funding (financial or material support) for your study. List the grants (with grant number) or organizations (with url) that supported your study, including funding received from your institution.  ii). State the initials, alongside each funding source, of each author to receive each grant. iii). State what role the funders took in the study. If the funders had no role in your study, please state: “The funders had no role in study design, data collection and analysis, decision to publish, or preparation of the manuscript.” iv). If any authors received a salary from any of your funders, please state which authors and which funders. | We modified the ‘Financial Disclosure statement’ in accordance to journal requirements. | - |
| 2. Please update the completed 'Competing Interests' statement. If you have no competing interests to declare, please state “The authors have declared that no competing interests exist”. | We modified our response to ‘Competing Interests’ as suggested. | - |
| 3. Please provide separate figure files in .tif or .eps format only and ensure that all files are under our size limit of 20MB. | We converted the figure to .tif format. | Figure |
| 4. We have noticed that you have uploaded supporting information but you have not included a list of legends.  Please add a full list of legends for all supporting information files (including figures, table and data files) after the references list. | We added a list of legends for supporting information after the references list. | Page 45 |
| 5. Please review your reference list to ensure that it is complete and correct. If you have cited papers that have been retracted, please include the rationale for doing so in the manuscript text, or remove these references and replace them with relevant current references. Any changes to the reference list should be mentioned in the rebuttal letter that accompanies your revised manuscript. If you need to cite a retracted article, indicate the article’s retracted status in the References list and also include a citation and full reference for the retraction notice. | We revised our reference list to make sure it is complete and correct. | Pages 37-45 |
| Reviewer 1 | | |
| 1. Data extraction and analysis (P. 9 to10): Include the name and scoring system of the quality assessment tool, as well as the determination of the quality of included studies. | Thanks for this comment. We named the scoring system of the quality assessment tool and stated how the scoring was determined.  Now it reads as follow:  *“The risk and sources of potential bias of each included study were assessed by two researchers (GS and GR) independently by using the Effective Public Health Practice Project Quality Assessment Tool (EPHPP) (22). This tool was developed to assess the quality of a diverse group of empirical studies. Each included study was assessed on study design, selection bias, confounders, blinding, data collection method, withdrawals and dropouts and assigned to either ‘strong’, ‘moderate’ or ‘weak’ category. Finally, the overall rating was determined based on these ratings as indicated in the assessment tool dictionary.”* | Page 7 |
| 2. Table 5 (P.31): Provide brief information related to the quality assessment tools, e.g. criteria to score the "selection bias". | We used the Effective Public Health Practice Project Quality Assessment Tool dictionary to score the selection bias. This dictionary is freely available (see the reference provided). Due to word limitation, we were not able to describe each criterion and its scoring. | - |
| 3. Table 5 (P.31): Include the sources of funding for the studies included in the review (this is one of the criteria of one of the assessment tools named AMSTAR 2 to evaluate the quality of the systematic review, once other authors conducted the systematic review of systematic reviews in the future. Relevant information can be found at: [https://amstar.ca/docs/AMSTAR-2.pdf;](https://protect-au.mimecast.com/s/6FTmC4QO8xS8y2WRUVAiZk?domain=amstar.ca) | Thanks for this comment, we will definitely consider using Amstar tool for future research when conducting systematic literature reviews. For this review we used the Effective Public Health Practice Project Quality Assessment Tool which does not require to list the sources of funding for the included studies. | - |
| 4. Results (P.10 to 30): Consider to analysis the effectiveness of interventions based on different countries or ethnicities, e.g. any countries have more significant results on the specific type of interventions. If possible. | This is a great suggestion and we initially aimed to do this analysis, however, majority of the included studies were from high income countries, therefore, we were unable to do the analysis based on different countries/ethnicities. We acknowledged this limitation in the limitations section. | - |
| Reviewer 2 | | |
| The review objective was clearly stated and appropriate inclusion criteria were defined. The review was limited to Published English language studies, therefore pertinent research in other languages and grey literatures have been missed. This has to be accounted in detail in the limitation. | We agree that we may missed some relevant literature published in other languages or grey literature. We acknowledged this limitation in the limitation section.  *“Also, this review was limited to the literature published in English language and did not included articles published in grey literature, therefore it may be we missed some important research written in other languages.”* | Pages 35-36 |
| The reported search strategy appeared to have missed index vocabularies (MeSH/ Emtree terms), hence difficult to judge how efficient it was. No additional attempts other than database search were made to locate more articles. | Senior research librarian at Macquarie University who has extensive experience in health research helped us to identify the keywords for this review and also helped us to identify the key databases suitable for public health and nutrition research. Therefore, we feel confident that we included sufficient number of databases and used appropriate keywords to identify studies. We reviewed over 5000 articles (see Prisma figure). | - |
| The synthesis included all relevant studies. Analyses pre-defined in the methodology section were performed appropriately. Synthesis was made in qualitative manner, due to this reason it is inappropriate to assess the heterogeneity and conduct the sensitivity analyses. However, inference has to be made based on study setting. Probably the review has be revised for high income countries. Including grey literatures could solve the skewed data for the high income countries. | Thank you for your comment. Unfortunately we were not able to assess the heterogeneity due to studies using different study designs and tools to measure outcomes. Majority of the included studies were from high income countries. We agree that we may missed some relevant research published in other languages. We acknowledged this as a limitation of our review and stated in the limitation section.  *“Also, this review was limited to the literature published in English language and did not included articles published in grey literature, therefore it may be we missed some important research written in other languages.”* | Pages 35-36 |
| The quality of the individual studies was not considered in the synthesis. | The methodological quality of majority studies were scored as ‘strong’ and ‘moderate’ quality, therefore, we did not considered it in the synthesis. However, the quality of each study is listed in Table 5. | Table 5 |
